# Metformin Alone and in Combinations Alter the Methylation Patterns of *ABCG1* and *TXNIP* Loci in Patients of Type 2 Diabetes

**DOI:** 10.2174/0118715303389767251009070040

**Published:** 2025-10-24

**Authors:** Shehla Shaheen, Shamim Mushtaq, Zahida Memon, Rubina Ghani, Asher Fawwad, Fatima Jehangir

**Affiliations:** 1 Department of Pharmacology, Ziauddin Medical College, Ziauddin University, Karachi, Pakistan;; 2 Department of Biochemistry, Ziauddin Medical College, Ziauddin University, Karachi, Pakistan;; 3 Department of Biochemistry, Jinnah Medical and Dental College, Sohail University, Karachi, Pakistan;; 4 Department of Basic Medical Sciences, Research and Diabetology, Baqai Institute of Diabetology and Endocrinology, Baqai Medical University, Karachi, Pakistan;; 5 Department of Family Medicine, Ziauddin Medical College and Ziauddin Hospital, Ziauddin University, Karachi, Pakistan

**Keywords:** DNA methylation, type 2 diabetes mellitus, glycemic parameters, precision medicine, body mass index, metformin

## Abstract

**Background:**

DNA methylation, being a predictor of gene-environment interaction, a dynamic and reversible process, and a target of drugs, may help clinicians to step towards precision medicine. Epigenome-wide association studies have linked methylation changes with type 2 diabetes and glycemic control; among such frequently documented differentially methylated loci include *TXNIP* (Thioredoxin interacting protein) and *ABCG1* (ATP-binding cassette Subfamily G Member 1). However, research evaluating the effects of antidiabetic treatment on DNA methylation is quite meager.

**Objective:**

The current study aimed to evaluate the pre-and post-treatment methylation status of *ABCG1* and *TXNIP* loci in individuals diagnosed recently with type 2 diabetes (T2Ds).

**Methods:**

In this quasi-experimental study, individuals recently diagnosed with T2Ds were recruited from 1^st^ March 2022 to 12^th^ June, 2023 from diabetes OPDS/clinics. We included the participants (total n=75) as groups that were prescribed Metformin (Met)alone (n=25), Metformin and Dipeptidyl Peptidase-4 inhibitors in combination (Met+DDP4I) (n=25), and Metformin and Sodium-Glucose co-Transporter 2 Inhibitors in combination (Met+SGLT2I) (n=25). The methylation status of *TXNIP* and *ABCG1* for all the study groups were evaluated by methylation-specific qPCR. Paired T-test and ANOVA were applied to compare the pre-and post-treatment methylation status of the study groups. Pearson’s correlation test followed by multiple linear regression analysis was performed to analyze the respective correlations and effect of different independent variables on the outcome of the study *i.e*., post-treatment methylation percentages of *ABCG1* and *TXNIP*.

**Results:**

In all groups, post-treatment *ABCG1* methylation was found to be significantly decreased, post-treatment *TXNIP* methylation displayed a significant increase. In a model of linear regression, among various dependent variables, BMI was observed to significantly influence post treatment *ABCG1* methylation in all groups, including Met alone (β=0.788, *p*=0.002), Met+DDP4I, (β= 0.754, *p*=0.04) and Met+SGLT2I (β= 0.733, *p*=0.027). While post-treatment TXNIP methylation was significantly affected by reduction in HbA1c levels in all groups, Met alone (β= -0.999, *p* <0.001), Met+DDP4I (β= -0.850 *p* <0.001) and Met+SGLT2I (β= -1.007, *p* <0.001).

**Conclusion:**

Metformin alone and its combinations with DDP4I and SGLT2I decrease the methylation of *ABCG1,* while increase the methylation of *TXNIP* in patients with type 2 diabetes. The post-treatment *ABCG1* methylation is associated with a decrease in BMI, whereas the post-treatment *TXNIP* methylation is associated with a decrease in HbA1c levels. Considering the effects of antidiabetic drugs on the methylation status of aforementioned loci involved in the control of glycemic and metabolic parameters; the results of the current study may pave a path for the implementation of precision medicine in type 2 diabetes after further validation by large scale clinical studies.

## 1 INTRODUCTION

Type 2 diabetes (T2D) is a complex entity of metabolic disorders involving a crosstalk between multiple genetic as well as environmental factors, including advancing age and lifestyle choices such as diet, body mass indices, physical activity, *etc*. [1-4]. Although extensive pharmacogenomic studies have explored multiple genetic aspects related to the variations in drug dosage, metabolism, therapeutic response, and risk of adverse effects among individuals through population-based genome-wide association studies, nevertheless the treatment of diabetes remains very complex and challenging due to the lack of feasibility in translating such research into clinical practice [5-7].

In recent years, the field of medicine has made remarkable advancements, giving rise to the concept of “Precision medicine” [8]. This approach helps in delivering customized treatments to the patients by considering their distinct characteristics, including genetic makeup, lifestyle, and environmental factors [9]. Hence, to streamline the transition of pharmacogenomics studies from research facilities to clinical settings, it is imperative to gain a thorough understanding of the gene-environment interactions associated with this multifaceted disease [10, 11].

DNA methylation is the most significant epigenetic event that controls gene expression as a consequence of the intricate interplay of genetic and non-genetic/environmental factors [12]. DNA methylation alterations can occur in response to biological, lifestyle, and environmental factors and play a vital role in normal cellular functions as well as in the development of diseases by altering the expression of genes [13, 14] Interestingly, methylation marks remain stable over time, yet they share the reversible quality of other epigenetic alterations. This combination of stability and reversibility not only enables easy detection of methylation changes but also highlights their potential as a target for therapeutic interventions [15, 16].

Methylation status of several genes linked to insulin resistance and T2D exhibited a significant correlation between tissues controlling glucose metabolism and peripheral blood [17]. Thus, estimation of DNA methylation of the genetic loci regulating glucometabolic pathways in blood may serve as surrogate markers owing to the convenience and non-invasiveness of sampling in comparison to the tissue sampling [18, 19]. Correspondingly, multiple longitudinal and retrospective Epigenome-wide association studies (EWAS) conducted on the blood samples of Type 2 diabetics have linked the methylation changes with T2D and glycemic parameters [20-22]. However, there is a scarcity of data related to the impact of antidiabetic treatment on DNA methylation.

So far, *TXNIP* (Thioredoxin interacting protein) and *ABCG1* (ATP-binding cassette Subfamily G Member 1) are among the few of the differentially methylated loci, frequently documented in T2Ds in comparison to controls, and have been associated with glycemic parameters including HbA1C, fasting blood sugar, fasting insulin levels and Homeostatic Model Assessment for Insulin Resistance (HOMA-IR) [23-26]. The *TXNIP* gene plays a critical role in pancreatic β-cell biology and diabetes development [27, 28]. Whereas *ABCG1* encodes a member of the ATP-binding cassette (ABC) protein family of transporters that facilitates the transport of glucose and lipids. The lack of this gene has been associated with impairment of glucose-stimulated insulin secretion and inflammation of pancreatic beta cells [29-31].

On the aforementioned grounds, we hypothesized that antidiabetic drugs may alter the aberrant methylation changes in *ABCG1* and *TXNIP* loci related to T2D and glycemic parameters. The current study aimed to evaluate the pre-and post-treatment methylation status of *ABCG1* and *TXNIP* loci in individuals diagnosed recently with type 2 diabetes (T2Ds) and who were prescribed metformin and its combinations with DDP4I and SGLT2I.

As of 2025, metformin is still the most frequently prescribed oral antidiabetic drug across the globe owing to its extraordinary antihyperglycemic potential and wide array of other beneficial metabolic effects attributed mainly to the activation of adenosine monophosphate-activated protein kinase (AMPK) [32, 33]. While recent research has explored some additional pathways linking metformin’s glucose-lowering effects with increased expulsion of glucose into the intestinal lumen, modification into the metabolites by gut microbiomes, and subsequent enhanced insulin sensitivity [34, 35]. Additionally, metformin improved glucose and lipid metabolism inside cardiomyocytes by deteriorating advanced glycation end products in the intestines [36] and offered renal [37-39], cardiovascular and retinal protective effects [40-43]. The contemporary research has focused on “metformin’s repurposing” owing to its wide spectrum of beneficial effects [44, 45], has exhibited significant anti-inflammatory and anti-oxidative potential in various cancers [46-49] and neuronal disorders [50, 51]. One of the review articles proposed that metformin, by virtue of the activation of AMP kinases, may augment the phosphorylation of enzymes involved in histone and DNA methylation and expression of microRNAs. These effects may contribute to the antidiabetic properties of metformin and underscores their potential to protect against cancers, cardiovascular diseases, cognitive impairment and process of ageing [52].

## 2 MATERIALS AND METHODS

### 2.1 Ethical Approval and Consents of the Study Participants

This study was approved by the “Ethical Review Committee of Ziauddin University” (reference no: 4200921- SSPHA). The study was conducted in accordance with the guidelines of the 1975 “Declaration of Helsinki”, as revised in 2013, and the ethical standards of the institutional and research committee. The participants were explained the methodology in detail and their written informed consents were obtained before the recruitment in the study. Written consents also include consents for their blood sampling, arthrometric measurements, and use of their samples and data for this research.

### 2.2 Study Design, Duration, and Settings of the Study

In this quasi-experimental study, individuals recently diagnosed with T2Ds were recruited from March 1, 2022, to June 12, 2023, from the OPDs of Baqai Institute of Diabetology and Endocrinology, Karachi, and Primary Health Care Clinics of Ziauddin Hospital, Karachi, Pakistan. At the same time, the benchwork was performed at the Multi-disciplinary Laboratories of Ziauddin University, Karachi, Pakistan.

### 2.3 Selection of the Study Participants, Sample Size Calculation, and Sampling Technique

The set inclusion criteria were both males and females (aged 30 to 70 years), recently diagnosed (within 3 months) with T2D, HbA1C in the range of 6.5% to 9% and who gave their written consents for participation in the study. The exclusion criteria were patients requiring insulin/ parenteral antidiabetic therapy or other drugs that affect the glycemic control, pregnant and lactating women, individuals unable to communicate efficiently or mentally unsound, having other endocrine disorders affecting glucose control, with a history of recent systemic infections, chronic kidney diseases, and alcohol addicts.

The study participants were selected by a non-probability convenience sampling technique [53-56]. The treatments were prescribed by the concerned diabetologist/clinician employing a patient-centered approach in accordance with the American Diabetes Association (ADA) “standards of care in diabetes” [57-59]. Recently, the American Diabetes Association and European Association for the Study of Diabetes have suggested the prioritized use of either SGLT2I, DPP4I, or Glucagon-like peptide-1 receptor agonist (GLP-1) in combination with Metformin if the therapeutic outcomes are insufficient with Metformin alone or to achieve glycemic targets earlier, employing a holistic and patient-centered approach in the management of T2D [58, 59]. Hence, in the current study, we included the participants as groups that were prescribed any of the following oral antidiabetic treatments as follows:

- Metformin (Met)alone
- Metformin and Dipeptidyl Peptidase- 4 inhibitors in combination (Met+DDP4I).
- Metformin and Sodium-Glucose co- Transporter 2 Inhibitors in combination (Met+SGLT2I).

The sample size of the study was estimated by the “sealed envelope power calculator” and was based on the post-treatment reductions in HbA1C with Metformin and sitagliptin (DDP4I), reported in the previous studies [60, 61]. The estimated sample size was 25 per group and hence a total of 75 for the three groups was considered (also approved by the institutional “Ethical Review Committee”).

### 2.4 Data Collection Procedure

All the study participants were provided with a detailed questionnaire to collect information related to their demographic characteristics. For the exercise status, moderate physical activity /brisk walk for at least 150 minutes/week was marked as “yes” [57], while smoking status was demarcated “yes” for the participants who had a history of smoking ≥100 cigarettes in their lifetime and continued to smoke at the time of induction in the study” [62]. Each participant of the study was called for a follow-up, 12 weeks after induction in the study, and was guided to report any health-related event during the entire period of the study in order to facilitate them. They were also advised for regular exercise /brisk walk and diet consumption according to the diabetic’s standard diet chart provided by the concerned clinician.

### 2.5 Anthropometric Measurements and Blood Collection

Anthropometric parameters include weight (in Kg) and height (in cms) measured *via* standard calibrated scales to calculate body mass index (BMI as Kg/m^2^), while waist circumference (WC in inches) was measured as per WHO standard guidelines [63]. All anthropometric measurements were taken in triplicate at baseline, and at 12 weeks follow-up for each study participant and the average of each of these measurements were used for the final analysis to minimize intra-observer variability. About 6 mL of venous blood was drawn by aseptic techniques from all the study participants after an overnight (at least 8 hours) both at the base line and after 12 weeks follow-up visists for the assessments of FBS and HbA1c and genomic DNA extractions.

### 2.6 DNA Extraction and Bisulphate Modification

Genomic DNA was extracted from 200 μl whole blood, performed by QIAamp DNA Mini kit (Qiagen), catalog no. 51306, as mentioned in the manufacturer’s manual. Quantification and purity of genomic DNA were assessed by the Multiskan sky-high microplate spectrophotometer (Thermo Fisher Scientific). Bisulphate modification of genomic DNA was carried out by EZ DNA Methylation-Gold Kit (ZYMO Research Corp. Catalog Number D5006). A total of 200-500 ng of input DNA in 20 μL volume was subjected to bisulfite conversion using a thermal cycler (Thermo Scientific Arktik, Fisher Scientific), under the following cyclic conditions: 98°C for 10 min, 64°C for 2.5 h and hold at 4°C (up to 20 h). About 10 µl of bisulfate-modified DNA was eluted in the kit provided elution buffer and stored at -80°C until further analysis.

### 2.7 Methylation-specific Primer Designing Tools

Primers specific for methylated and unmethylated DNA sequences were designed using MethPrimer version 2.0 (http://www.urogene.org/methprimer2/). For methylation-specific PCR, two pairs of primers were designed, one of them was specific for modified and methylated DNA (M-Forward and Reverse), and the other for modified and unmethylated DNA (U Forward and Reverse). While DAPK1 primers (Zymo Cat no. D5014) were used as an internal control of the process (Table **1**).

### 2.8 Methylation Specific qPCR and Methylation Analysis

One microliter (1-μl) of bisulphite modified DNA was added to 50 μL PCR reaction mixture containing 25 μl of qPCR premix Zymo*Taq* (ZYMO Research Corp Cat. No E2055.), and 1 µl of each forward and reverse primer (10 µM) was added to make a total volume of 50µl according to the manufacturer’s instructions. The PCR amplification was performed under the following cyclic conditions, initial denaturation at 95°C for 10 min; followed by 30-40 cycles of denaturation at 95°C for 30 s, primers annealing at optimized temperatures (range 55-60°C) for 30-40 s, and extension at 72°C for 30-60 s; with a final extension at 72°C for 7 min. Two separate cycles were performed for methylated and unmethylated set of primers using Aria Mx Real-time qPCR instrument and system (Agilent). For the internal control, a human methylated & non-methylated DNA set provided with control primers, targeting the DAPK1 (Zymo Cat no. D5014) gene was used in each run according to the manual instructions. Each sample was analyzed in duplicate for both methylated and un-methylated reactions.

The methylation percentages of each *ABCG1* and *TXNIP* were calculated by the following equation (64):

Methylation percentage (%) = 100/[1+2DCt(methylated-unmethylated)] %

DCt (methylated-unmethylated) was calculated for each *ABCG_1_* and *TXNIP* by subtracting the Ct values of methylated *ABCG1* and *TXNIP* signals from the respective Ct values of the unmethylated *ABCG1* and *TXNIP* signals.

### 2.9 Statistical Analysis

Statistical Package for the Social Sciences (SPSS) version 24.0 was used for data entry and analysis. The normality of the data was determined by Levene’s and Shapiro-Wilk tests. Numeric variables were expressed as mean and standard deviation, while categorical variables were expressed as frequencies and percentages. Paired T test was applied to estimate pre- and post-treatment changes among all groups, while ANOVA followed by post-hoc Tukey’s test was applied for intergroup comparisons. The correlation of different variables with post-treatment methylation status was analyzed by Pearson’s correlation test. To analyze the impact of the study’s parameters on the outcome of the study (post-treatment methylation % of *ABCG1* and *TXNIP*), multiple linear regression analysis was performed. In the model of multiple linear regression, age, BMI, WC, FBS, HbA1c, smoking history, and exercise status were independent variables, while post-treatment methylation percentages of *ABCG1* and *TXNIP* were taken as dependent variables. A *p*- value of <0.05 was considered to be significant.

## 3 RESULTS

### 3.1 Process of Selection and Characteristics of the Study Participants

**Fig. (1):**
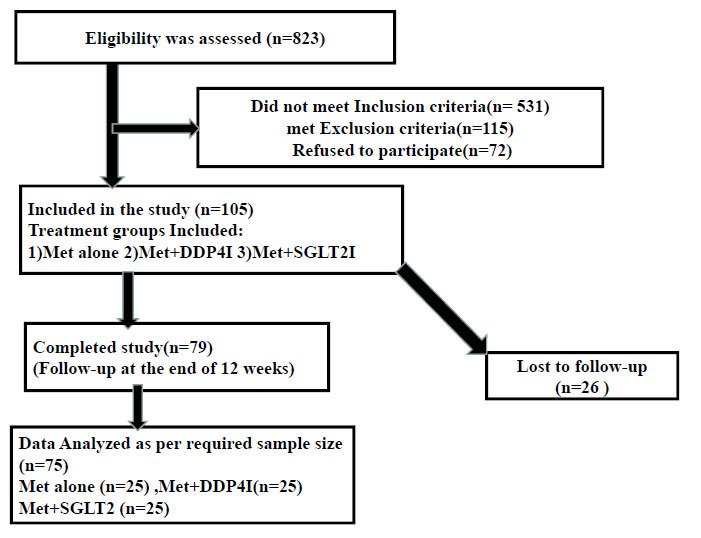
Flow diagram of the process of selection of the study participants.

Individuals recently diagnosed with T2Ds, visiting the OPD/clinics from 1^st^ March 2022 to 12^th^ June 2023, were interviewed for their eligibility(n=823). Among those, 105 met the inclusion criteria and were included in the study. Although the target sample size was 75, but additional 30 participants were included in order to account for the lost to follow-up. The selected participants were grouped into three groups based on their respective prescribed treatments (Met alone, Met +DDP4I, and Met+SGLT2I). In the first two weeks, mild GIT upsets were reported among a few of the study participants (Met alone=5, Met+DDP4I=2, MeT+SGLT2I=2), however, these symptoms resolved as the patients adapted to the treatments with passing time. Out of the selected,79 participants completed the study and came for follow-up at 12 weeks. The follow-up of the last included participant ended on 12^th^ September 2023, and finally data of 75 study participants were analyzed as per the estimated sample size (Fig. **1**).

The main characteristics of the study groups, including age, gender, smoking, history and exercise status, are displayed in Supplementary File **1**.

### 3.2 Glycemic and Anthropometric Parameters

The paired differences between all respective pre-treatment and post-treatment anthropometric parameters were highly significant for all treatment groups (*p* <0.001). While intergroup comparisons for pretreatment and post-treatment anthropometric parameters, BMI and WC, were not significant (Table **2**). The paired comparisons for both HbA1C and FBS have shown a highly significant (*p* <0.001) reductions after their respective treatments. Significant differences were found after multiple comparisons of various groups for post-treatment HbA1c (*p* =0.03) and FBS (*p*=0.002). Post Hoc analysis revealed significant differences for HbA1c between Met alone and Met+SGLT2I (*p*=0.042) and for FBS between Met alone and Met+DDP4I (*p*=0.004) and between Met alone and Met+ SGLT2I (*p*=0.013)(represented in parentheses, Table **2**).

### 3.3 Methylation Status of Genetic Loci

**Fig. (2):**
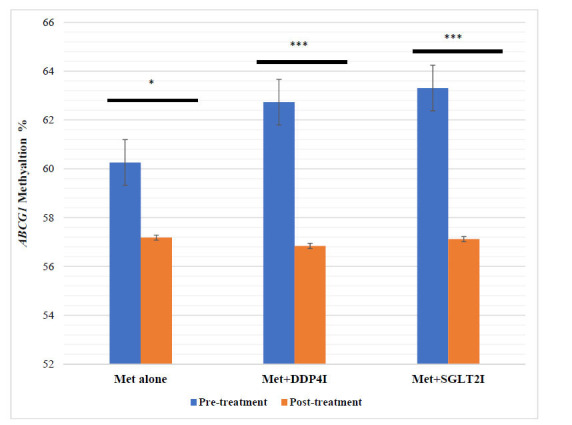
Pre and post-treatment methylation status (%) of *ABCG1*. Y-axis shows *ABCG1* Methylation percentages for the treatment groups (n=25 each), represented in the x-axis as Metformin (Met) alone, Metformin and Dipeptidyl Peptidase- 4 inhibitors in combination (Met+DDP4I), and Metformin and Sodium-Glucose co-Transporter 2 Inhibitors in combination (Met+SGLT2I). Significance levels are denoted by p-value as follows: (*≤0.05) and (***≤ 0.001), estimated by paired t-test for intragroup comparisons of methylation status.

An overall post-treatment reduction of *ABCG1* methylation percent (%) was observed in all groups; the paired differences between pretreatment and post-treatment *ABCG1* methylation (%) are shown to be significant in Met alone (*p*=0.016), Met+DDP4I (*p*<0.001), and Met+SGLT2I (*p*<0.001) (Fig. **2** and Supplementary File **2**). The amplification curves of the respective methylated and unmethylated *ABCG1* are embodied in Supplementary File **3**.

**Fig. (3):**
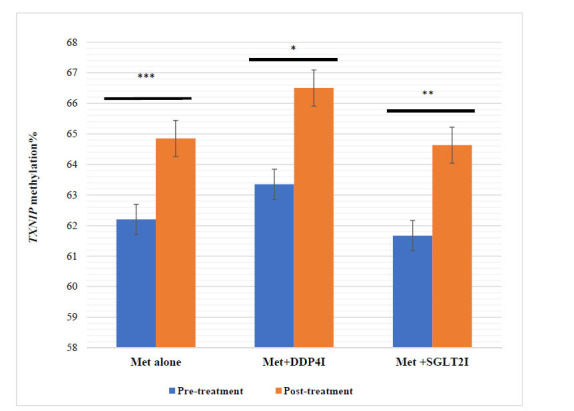
Pre-and post- treatment methylation status (%) of *TXNIP*. Y-axis shows *TXNIP* Methylation percentages for the treatment groups (n=25 each), represented in the x-axis as Metformin (Met)alone, Metformin and Dipeptidyl Peptidase- 4 inhibitors in combination (Met+DDP4I), and Metformin and Sodium-Glucose Co-Transporter 2 Inhibitors in combination (Met+SGLT2I). Significance levels are represented by p-value as follows: (*≤0.05), (**≤0.005), and (*** ≤0.001), estimated by paired t-test for intragroup comparisons of methylation status.

The post-treatment *TXNIP* methylation percentages in all groups were found to be decreased, along with the paired differences between pretreatment and post-treatment *TXNIP* methylation % shown to be highly significant in Met alone (*p*<0.001) and significant in Met+ DDP4I (*p*= 0.007) and Met+SGLT2I (*p*=0.002) groups (Fig. **3** and Supplementary File **2**). The amplification curves of the respective methylated and unmethylated *TXNIP* are embodied in Supplementary File **3**.

Post-treatment *ABCG1* methylation has positive and highly significant correlations with BMI (*p*-values <0.001) and with WC (*p*-values <0.001) in all treatment groups. Also, the correlation of *ABCG1* methylation% with HbA1C is shown to be positive and highly significant in met+DDP4I (*p*=0.001), met alone (*p*=0.007), and met+SGLT2I (*p*=0.03) groups. *ABCG1* methylation % and FBS are also shown to have positive and significant correlations in met+DDP4 (*p*=0.003), and met+SGLT2I (*p*=0.03) groups (Table **3**).

On the contrary, the post-treatment *TXNIP* methylation has highly significant negative correlations with HbA1C (*p*=0.001) in all treatment groups. Correlations of *TXNIP* are also inverse and highly significant with FBS in met+SGLT2I (*p*=0.001) and met+DDP4I (*p*=0.001) groups. Regarding post-treatment *TXNIP* methylation and BMI, negative and highly significant correlations are observed in met+DDP4I (*p*=0.001), significant in met alone (*p*=0.007), and met+SGLT2I (*p*=0.021) groups. Similarly, WC and post-treatment methylation % are found to have a negative and highly significant correlation in met+DDP4I (*p* = 0.001), significant in met alone (*p*=0.002), and met+SGLT2I (*p* = 0.018) groups (Table **3**).

In the multiple linear regression model of age, HbA1C, FBS, BMI, WC, exercise status and smoking history as independent variables and post-treatment *ABCG1* methylation as a dependent variable, only BMI was observed to have a significant impact on the post treatment *ABCG1* methylation in all groups, Met alone (β=0.788, *p*=0.002), Met+DDP4I, (β= 0.754, *p*=0.04) and Met+SGLT2I (β= 0.733, *p*=0.027) (Table **4**).

In the multiple linear regression model of age, HbA1C, FBS, BMI, WC, exercise status and smoking history as independent variables and post-treatment methylation status of *TXNIP* as the dependent variable; only HbA1c was found to have a significant influence on the post-treatment *TXNIP* methylation in all groups, Met alone (β= -0.999, *p* <0.001), Met+DDP4I (β= -0.850 *p* <0.001), and Met+SGLT2I (β=-1.007, *p* <0.001) (Table **5**).

## 4 DISCUSSION

The current study has reported for the first time the effects of metformin alone and combinations (Met+DDP4I and Met+SGLT2I) on the methylation status of *ABCG1* and *TXNIP* loci in the same individuals with T2D. A significant reduction of *ABCG1* methylation was observed in all groups, including metformin alone and its combinations (*p* <0.001) (Fig. **2**, Supplementary File **2**). Consistent with the post-treatment decrease in the methylation of *ABC*G_1_, significant reductions in both glycemic parameters, HbA1c and FBS have been shown in all treatment groups (p values<0.00) (Table **2**). Various EWAS conducted on the peripheral blood have documented the association of increased *ABCG1* methylation with impaired glycemic control and have been reported to be prevalent in individuals with T2D. Moreover, EWAS conducted on the diverse populations supported that *ABCG1* hypermethylation has been positively associated with FBS, fasting insulin, and HOMA-IR in T2Ds and controls [65-67]. We suggest that the post-treatment reduction in *ABCG1* methylation observed in all groups has potentially increased the genetic expression of *ABCG1,* which has subsequently improved the glycemic parameters observed in all of our study groups. As previously documented, the hypermethylation of *ABCG1* in the blood was found to be reproducibly associated with its reduced gene expression, along with higher levels of HbA1c and fasting insulin levels in the same study subjects [29]. Similarly, in comparison to controls, Chambers *et al*., have also discovered increased methylation of *ABCG1* along with the decreased gene expression in the blood of T2Ds among Asians and Europeans [26] More importantly, *ABCG1* encodes a member of the ATP-binding cassette (ABC) protein family of transporters that are involved in the removal of surplus cholesterol from peripheral tissues to the liver and manages the homeostatic control of glucose and lipids [30] * ABCG1* also augments insulin secretion *via* HDL, the lack of both *ABCG1*, and another related transporter, *ABCA1* is followed by the accumulation of sterols, hampered glucose-stimulated insulin secretion as well as pancreatic beta cells inflammation [30, 31].

We found a significant increase in post-treatment *TXNIP* methylation in all of the study groups, including metformin alone (*P*- value <0.001), metformin+ DDP4I (*P*-value <0.007), and SGLT2I (*P*-value <0.002) (Fig. **3**, Supplementary File **2**). Furthermore, along with the post-treatment increase in the methylation of *TXNIP*, we observed significant reductions in both glycemic parameters, HbA1c and FBS, after treatment among all groups (p values<0.001) (Table **2**). Parallel to our findings, in a recent research, a higher regional DNA methylation level at *TXNIP* was significantly associated with lower fasting glucose, HbA1c, and HOMA-IR. Moreover, after consuming an average-protein weight-loss diet, the participants with higher *TXNIP* methylation levels displayed greater reductions in insulin and HOMA-IR levels, regardless of weight loss [68]. We suggest that post-treatment increase in *TXNIP* methylation has reduced its genetic expression with the subsequent reduction in glycemia. The finding of this study is supported by the previous reports; in one of such study, deranged *TXNIP* methylation was regarded as a predictor of abnormal glucose homeostasis. Furthermore, hypomethylation of DNA at *TXNIP* led to induce gene expression and was repetitively linked with higher glucose levels, decreased functional capacity of insulin-secreting cells, increased insulin resistance, and T2D [68]. Correspondingly, previous EWAS have also documented the correlation of decreased methylation of *TXNIP* with higher FBS, HbA1c, and HOMA-IR in T2D patients [65, 66, 69]. It has been highlighted that increased expression of *TXNIP* facilitates glucotoxicity-mediated β-cell death, while absence or deficiency of *TXNIP* helps to improve β-cell survival and decrease the risk of TD1 and T2D [70, 71]. However, it cannot be proposed whether increased *TXNIP* after treatment is a cause or an effect of the reduction in glycemia, since hyperglycemia was shown to increase *TXNIP* expression in various cells and tissues [26, 72]. High glucose levels induce *TXNIP* expression *via* activation of the carbohydrate response element-binding protein and downregulate *GLUT1,* a major transmembrane glucose transporter across the cell [73]. These findings led to the idea that suppression of *TXNIP* in prediabetic and diabetic conditions may be beneficial for treating human diabetes [70, 71].

Despite extensive documentation of methylation marks in type 2 diabetes and their associations with the glycemic parameters, there is insufficient data related to the impact of antidiabetic drugs on the methylation markers, especially those associated with glycemic control. Among few of the DNA methylation studies conducted on type 2 diabetes treatment so far, the most frequently prescribed anti-diabetic drug, metformin was shown to decrease DNA methylation at promoters of its transporter genes *SLC22A1, SLC22A3*, and *SLC47A1* [74]. Metformin treatment for a longer period showed distinct methylation regions in the blood of individuals having T2D, in contrast to 12 drug-naïve T2Ds [75]. Furthermore a research proposed GLP-1 agonists to be the substantial predictor of *NF-κB* DNA methylation in individuals with T2D [76].

Being a component of a complex metabolic syndrome, T2D and its manifestations are associated with obesity, frequently assessed by BMI calculations (based on height and weight measurements); additionally, WC measurements are performed for the assessment of central obesity [77]. In this study, significant reductions in both post-treatment anthropometric parameters, BMI and WC have been seen in comparison to their pretreatment levels in all study groups (*P*-values <0.001, Table **2**). We found that post-treatment *ABCG1* methylation has significant positive correlations with HbA1c, FBS, BMI, and WC in all groups (Table **3**). On the contrary, we observed that post-treatment *TXNIP* methylation has inverse correlations with BMI, WC, HbA1c, and FBS in all groups (Table **3**). To eliminate the effects of confounders, we performed further analysis to observe the impact of various parameters, age, HbA1c, FBS, BMI, WC, exercise status, and smoking history on the outcome of the study, the post-treatment methylation status of *ABCG1* (Table **4**) and *TXNIP* (Table **5**). We observed that after adjusting for age, BMI, WC, HbA1c, smoking history, and exercise status, only BMI was found to be significantly affecting the post-treatment *ABCG1* methylation in all groups (Table **4**). Consistent with our findings, previous EWAS conducted on T2Ds have reported the correlations of BMI and *ABCG1* methylation, so the alterations in *ABCG1* methylation were attributed to the changes in lipid and glucose metabolism as a result of BMI modifications [24, 29]. It has already been emphasized that BMI reduction is associated with improved glycemic control and is a cornerstone in the management and prevention of T2D [78]. Furthermore, in a longitudinal EWAS conducted on individuals with overweight and obesity, the study participants having lower basal *ABCG1* methylation were observed to achieve significantly more reductions in weight, WC, and central adiposity after dietary interventions [79]. Regarding post-treatment *TXNIP* methylation, on further evaluation, we found that only HbA1c was significantly influencing the post-treatment *TXNIP* methylation status after adjusting for various factors such as age, BMI, WC, FBS, smoking history, and exercise status in all groups (Table **5**). Parallel to our study results, Meeks *et.al.,* explored that the correlations of *TXNIP* and HbA1c remained significant after adjusting for BMI and other factors in T2D individuals [80].

On these grounds, we suggest that metformin and its combinations with DDP4I and SGLT2I mediate their effects on *ABCG1* and *TXNIP* by altering BMI and HbA1c, respectively. This could be additional but indirect effects of metformin and its combinations besides their well-known effects on glycemic control and BMI in the management of T2D.

### 4.1 Strengths and Limitations

To the best of our knowledge, this study is innovative as for the first time the pre and post treatment effects of metformin alone and its combinations with DDP4I and SGLT2I were evaluated on the methylation status of *ABCG1* and *TXNIP* by a direct comparison in the same patients with T2D. We also inspected the bearing of various demographic factors, including age, BMI, WC and lifestyle factors such as exercise and smoking status, on the post-treatment methylation status of *ABCG1* and *TXNIP* loci in all groups to validate our findings and to understand the complex interactions of various factors affecting the outcome of the treatment of T2D.

The limitation of the study was that the effects of antidiabetic drugs on the methylation status of *ABCG1* and *TXNIP* loci were evaluated for a short duration (12 weeks for each study participant). Also, the study was conducted in a subset of a single population and with a relatively small sample size, therefore may affect the generalization. Furthermore, we were not able to assess the impact of diet which might be a potential confounding factor in this study.

## CONCLUSION

We conclude that metformin alone and its combinations with DDP4I and SGLT2I decrease the methylation of *ABCG1,* while increase the methylation of *TXNIP* in individuals with type 2 diabetes. The post-treatment *ABCG1* methylation is correlated with the reduction in BMI, whereas the post-treatment *TXNIP* methylation is correlated with the decrease in HbA1c levels. Moreover, HbA1C is a predictor of the post-treatment methylation status of *TXNIP,* whereas BMI is a predictor of the post-treatment methylation status of *ABCGI*. Further validation by large-scale clinical studies is required to dissect out the role of antidiabetic drugs on DNA methylation markers and their association with glycemic and other metabolic parameters to optimize the management of type 2 diabetes.

## AUTHORS' CONTRIBUTIONS

The authors confirm their contribution to the paper as follows: SS: Study concept and design, Data analysis and interpretation, Writing the paper, Writing-review and editing. S.M. methodology, Data validation and investigation, Writing-review, and editing, project supervision. Z.M., writing-review and editing, Project supervision and administration, R.G., data curation, visualization, writing-review, and editing A.F., writing-review and editing, project supervision and administration, F.J., data collection, data curation, writing-review, and editing. All authors read and approved the final manuscript.

## Figures and Tables

**Table 1:** Sequences of methylated and unmethylated primers for the *TXNIP* and *ABCG1* genes.

**Gene Name**	**Accession No.**	**Primers Sequence (5' to 3')**	**Product Size**
***ABCG1***	NM_ 01681 8.3	M-Forward- GTAGTTGTAGTTTCGAGCGG	89
		M-Reverse- GCCGAAATTTACATAACCCG	
		U- Forward- AGTTGTAGTTTTGAGTGGGG	89
		U-Reverse-ACACCAAAATTTACATAACCCAAAT	
***TXNIP***	XM_ 017000085	M-Forward- GATTTTGAAAAGGTGTACGGT	186
		M-Reverse- AAAAAACGTATCTTCATAACGCAA	
		U- Forward -GATTTTGAAAAGGTGTATGGTA	184
		U-Reverse- AAAACATATCTTCATAACACAAATACTCC	
**DAPK1 (Control Primers)**	-	Forward -TAGAATTTAGTTAGAGGGTAGTTTAGTA	
		Reverse- AAACRACCAATAAAAACCCTACAAA	

**Table 2:** Comparison of groups for glycemic and anthropometric parameters.

**Groups**	**BMI(Kg/m^2^)**			**WC (inches)**			**HbA1c (%)**			**FBS (mg/dL)**		
	**Pre-treatment**	**Post-treatment**	***p*-value**	**Pre-treatment**	**Post-treatment**	***p*-value**	**Pre-treatment**	**Post-treatment**	***p*-value**	**Pre-treatment**	**Post-treatment**	***p*-value**
**Met alone (n=25)**	28.782 ± 4.469	28.039 ± 4.157	0.001 ***	39.42 ± 3.53	37.952 ± 3.345	0.001 ***	7.480 ± 0.510	6.420 ± 0.563 (a)	0.001 ***	225.32 ± 60.52	159.28 ± 33.31 (a), (b)	0.001 ***
**Met+ DDP_4_I (n=25)**	28.616 ± 4.229	27.980 ± 4.265	0.001 ***	38.656 ± 3.578	37.428 ± 3.289	0.001 ***	7.628 ± 0.670	6.156 ± 0.3743	0.001 ***	235.72 ± 46.74	136.72 ± 15.14 (b)	0.001 ***
**Met+ SGLT_2_I (n=25)**	30.824 ± 6.196	29.844 ± 5.856	0.001 ***	40.084 ± 2.965	38.560 ± 2.533	0.001 ***	7.52 ± 0.59	6.12 ± 0.26 (a)	0.001 ***	226.32 ± 53.93	139.44 ± 19.78 (a)	0.001 ***
***p*-value n=75**	0.233	0.305	-	0.331	0.433	-	0.66	0.030 *	-	0.755	0.002 **	-

**Abbreviations:** Body mass index (BMI), waist circumference (WC), Glycosylated hemoglobin (HbA1c), Fasting blood sugar (FBS) are expressed as mean ± SD. Significance levels are represented by p-value as follows: (* ≤0.05), (** ≤0.005), and (*** ≤0.001), estimated by paired t-test and ANOVA for intra-group and inter-group comparisons, respectively. Post-hoc Tukey’s test significance for multiple comparisons is expressed between groups as: (a) Met alone and Met+SGLT2I (b)Met alone and Met+DDP4I

**Table 3:** Correlations of post-treatment *ABCG1* and *TXNIP* methylation % with various parameters in treatment groups.

-	**Post-treatment *ABCG1* Methylation (%)**		
**Variables**	**Met alone (n=25) ** **r (*p*- value)**	**Met+DDP4I (n=25) ** **r (*p*-value)**	**Met+SGLT2 I (n=25) ** **r (*p*-value)**
Age	-0.249(0.22)	-0.193(0.35)	-0.061(0.77)
HbA1C%	0.527 (0.007) **	0.712 (0.001) ***	0.417 (0.03) *
FBS	0.310 (0.13)	0.571 (0.003) **	0.434 (0.03) *
BMI	0.903 (0.001) ***	0.937 (0.001) ***	0.935 (0.001) ***
WC	0.840 (0.001) ***	0.909 (0.001) ***	0.935 (0.001) ***
	**Post-treatment *TXNIP* Methylation (%)**		
Age	-0.036 (0.86)	0.267 (0.197)	0.265 (0.20)
HbA1C%	-0.940 (0.001) ***	-0.957 (0.001) ***	-0.916 (0.001) ***
FBS	-0.288 (0.16)	-0.613 (0.001) ***	-0.863 (0.001) ***
BMI	-0.528 (0.007) **	-0.786 (0.001) ***	-0.458 (0.021) *
WC	-0.595 (0.002) **	-0.734 (0.001) ***	-0.468 (0.018) *

**Note: ** Pearson’s correlation test was applied, r= Coefficient of correlation, 2-tailed significance levels represented in parentheses as p values are as follows (* ≤ 0.05), (**≤ 0.005), and (***≤ 0.001).

**Table 4:** Regression analysis for the post-treatment methylation status (%) of *ABCG1*.

	**Post-treatment *ABCG1* methylation (%) (Met alone, n=25)**					
**Variables**	**B**	**Std. Error**	**β**	**95% CI**		***p***
				**LB**	**UB**	
Age	-0.017	0.030	-0.051	-0.079	0.046	0.579
HbA1c	0.151	0.816	0.023	-1.570	1.872	0.855
FBS	0.006	0.006	0.112	-0.006	0.019	0.293
BMI	0.599	0.159	0.788	0.263	0.935	0.002**
WC	-0.046	0.214	-0.047	-0.497	0.406	0.834
Smoking history	0.819	0.694	0.123	-0.644	2.282	0.254
Exercise status	-2.078	0.789	-0.267	-3.743	-0.414	0.017
	**Post-treatment *ABCG1* methylation (%) (Met+DDP4I, n=25)**					
**Variables**	**B**	**Std. Error**	**β**	**95% CI**		***p***
				**LB**	**UB**	
Age	0.034	0.023	0.127	-0.014	0.082	0.155
HbA1c	-0.105	0.543	-0.025	-1.251	1.041	0.849
FBS	0.003	0.006	0.055	0-.009	0.016	0.582
BMI	0.507	0.230	0.754	0.022	0.992	0.041*
WC	0.156	0.261	0.197	-0.395	0.708	0.558
Smoking history	-0.806	0.496	-0.144	-1.852	0.241	0.123
Exercise status	-0.409	0.572	-0.059	0.798	0.484
	**Post-treatment *ABCG1* methylation (%) (Met+SGLT2I, n=25)**					
**Variables**	**B**	**Std. Error**	**β**	**95% CI**		***p***
				LB	UB	
Age	-0.041	.035	-0.098	-0.114	0.032	0.250
HbA1c	1.161	1.108	0.211	-1.177	3.500	0.309
FBS	-0.007	0.012	-0.121	-0.033	0.019	0.565
BMI	0.379	0.157	0.733	0.048	0.711	0.027*
WC	0.225	0.322	0.208	-0.454	0.904	0.494
Smoking history	-0.406	0.479	-0.065	-1.417	0.606	0.409
Exercise status	-0.448	0.581	-0.057	-1.673	0.777	0.451

**Abbreviations:** B = Unstandardized coefficient, SE=Standard Error, β = Standardized coefficient, CI = Confidence Interval, LB and UB = lower and upper boundaries. Significance levels represented as *p* are as follows: (* ≤ 0.05) and (**≤0.005).

**Table 5:** Regression analysis for the post-treatment methylation status (%) of *TXNIP*.

	**Post-treatment *TXNIP* methylation (%) (Met alone, n=25)**					
**Variables**	**B**	**Std. Error**	**β**	**95% CI**		***P***
				**LB**	**UB**	
Age	-0.020	0.027	-0.057	-.077	0.037	0.472
HbA1c	-6.905	0.744	-0.999	-8.476	-5.335	0.001***
FBS	0.007	0.005	0.119	-0.004	0.018	0.197
BMI	0.134	0.145	0.166	-0.173	0.440	0.370
WC	-0.107	0.195	-0.105	-0.519	0.306	0.592
Smoking history	0.621	0.633	0.088	-0.715	1.957	0.340
Exercise status	0.586	0.720	0.071	-0.933	2.105	0.427
	**Post-treatment *TXNIP* methylation (%) (Met + DDP4I, n=25)**					
**Variables**	**B**	**Std. Error**	**β**	**95% CI**		***P***
				**LB**	**UB**	
Age	-0.019	0.037	-0.035	-0.097	0.060	0.621
HbA1c	-7.365	0.882	-0.850	-9.225	-5.504	0.001***
FBS	-0.011	0.010	-0.089	-0.031	0.009	0.267
	**Post-treatment *TXNIP* methylation (%) (Met + DDP4I, n=25)**					
**Variables**	**B**	**Std. Error**	**β**	**95% CI**		***P***
				**LB**	**UB**	
BMI	-0.608	0.373	-0.446	-1.395	0.179	0.122
WC	0.605	0.424	0.376	-0.290	1.501	0.172
Smoking history	0.229	0.805	0.020	-1.470	1.928	0.780
Exercise status	-0.055	0.929	-0.004	-2.016	1.905	0.953
	**Post-treatment methylation *TXNIP* (%) (Met + SGLT2I, n=25)**					
**Variables**	**B**	**Std. Error**	**β**	**95% CI**		***P***
				**LB**	**UB**	
Age	-0.064	0.042	-0.142	-0.153	0.026	0.152
HbA1c	-5.884	1.358	-1.007	-8.748	-3.019	0.001***
FBS	0.006	0.015	0.103	-0.025	0.038	0.672
BMI	0.120	0.192	0.218	-0.286	0.526	0.542
WC	-0.459	0.394	-0.399	-1.291	0.373	0.260
Smoking history	-0.244	0.587	-0.037	-1.483	0.995	0.683
Exercise status	-0.857	0.711	-0.103	-2.358	0.643	0.245

**Abbreviations:** B= Unstandardized coefficient, SE = Standard Error, β = Standardized coefficient, CI = Confidence Interval, LB and UB = lower and upper boundaries, Significance levels represented as *p* are as follows: (*** ≤ 0.001).

## Data Availability

The authors confirm that the data supporting the findings of this study are available within the article and its supplementary materials.

## LIST OF ABBREVIATIONS

TXNIP: Thioredoxin Interacting Protein
ABCG1: ATP-binding Cassette Subfamily G Member 1
T2Ds: Type 2 Diabetes
ABC: ATP-binding Cassette
AMPK: adenosine monophosphate-activated protein kinase
ADA: American Diabetes Association
GLP-1: Glucagon-like peptide-1

## STANDARDS OF REPORTING

The “TREND statement-reporting ”guidelines were followed.
