# Supplementary material for: Metformin Alone and in Combinations Alter the Methylation Patterns of ABCG1 and TXNIP Loci in Patients of Type 2 Diabetes
Source: Endocr Metab Immune Disord Drug Targets. 2025 Oct 24;26:E18715303389767. doi: 10.2174/0118715303389767251009070040 (PMC13284670; doi:10.2174/0118715303389767251009070040)
Supplement: Supplementary file 1 [file EMIDDT-26-E18715303389767_SD1.pdf]

Supplementary Material

Metformin Alone and in Combinations Alter the Methylation Patterns of ABCG1 and TXNIP Loci in Patients of Type 2 Diabetes

Shehla Shaheen[1,*], Shamim Mushtaq[2], Zahida Memon[1], Rubina Ghani[3], Asher Fawwad[4] and Fatima Jehangir[5]

[1]Department of Pharmacology, Ziauddin Medical College, Ziauddin University, Karachi, Pakistan; [2]Department of Biochemistry, Ziauddin Medical College, Ziauddin University, Karachi, Pakistan; [3]Department of Biochemistry, Jinnah Medical and Dental College, Sohail University, Karachi, Pakistan; [4]Department of Basic Medical Sciences, Research and Diabetology, Baqai Institute of Diabetology and Endocrinology, Baqai Medical University, Karachi, Pakistan; [5]Department of Family Medicine, Ziauddin Medical College and Ziauddin Hospital, Ziauddin University Karachi, Pakistan

Supplementary File 1: Characteristics of the study groups.

**Parameters**	**Met alone (n=25)**		**Met+DDP4I(n=25)**		**Met+SGLT2I(n=25)**	
**Age (years) mean ± SD**	49.48 ± 10.21		48.92 ± 10.66		50.80 ± 7.62	
**Gender n (%)**	Male	Female	Male	Female	Male	Female
	13(52%)	12(48%)	15(60%)	10(40%)	14 (56%)	11(44%)
**Smoking history n (%)**	Yes	No	Yes	No	Yes	No
	11(44%)	14(56%)	12(48%)	13(52%)	11(44%)	14(56%)
**Exercise status n (%)**	Yes	No	Yes	No	Yes	No
	6(24%)	19(76%)	5 (20%)	20(80%)	5 (20%)	20(80%)

The base line characteristics of the study groups including age is expressed as mean ±SD, while gender, smoking and exercise are expressed as n (%).

Supplementary File 2: Comparison of pretreatment and post treatment Methylation status (%) of *ABCG1* &*TXNIP* for study groups.

Drug Group	ABCG1 Methylation (%)			TXNIP Methylation (%)		
	**Pre-treatment**	**Post treatment**	**P-value**	**Pre-treatment**	**Post-treatment**	**P-value**
**Met alone (n=25)**	60.26±4.80 (*)	57.18 ± 3.40	0.016*	62.20±3.51	64.85±3.59	0.001***
**Met+DDP$_4$I (n=25)**	62.73±4.23	56.84 ± 2.83	0.001***	63.35 ±4.41	66.50±5.76	0.007**
**Met+SGLT$_2$ I(n=25)**	63.31±4.03 (*)	57.12±3.20	0.001***	61.67± 3.03	64.63±3.40	0.002**
**P-value n=75**	0.038*	0.992		0.268	0.266	

There is an overall post-treatment reduction of *ABCG1* methylation percent (%) in all groups, the paired differences between pretreatment and post-treatment *ABCG1* methylation (%) are shown to be significant in Met alone (*p*=0.016), Met+DDP4I(*p*<0.001) and Met+SGLT2I (*p*<0.001). Also display an overall increase in the post-treatment *TXNIP* methylation % in all groups, the paired differences between pre-treatment and post-treatment. *TXNIP* methylation % are observed to be highly significant in Met alone (*p*<0.001) and significant in Met+ DDP4I(*p*= 0.007) and Met+SGLT2I (p=0.002)groups. Intergroup comparison by ANOVA is slightly significant only for pretreatment *ABCG1* between Met alone and Met+SGLT2I(p=0.038), while intergroup differences for post treatment *ABCG1* and *TXNIP* are non-significant.

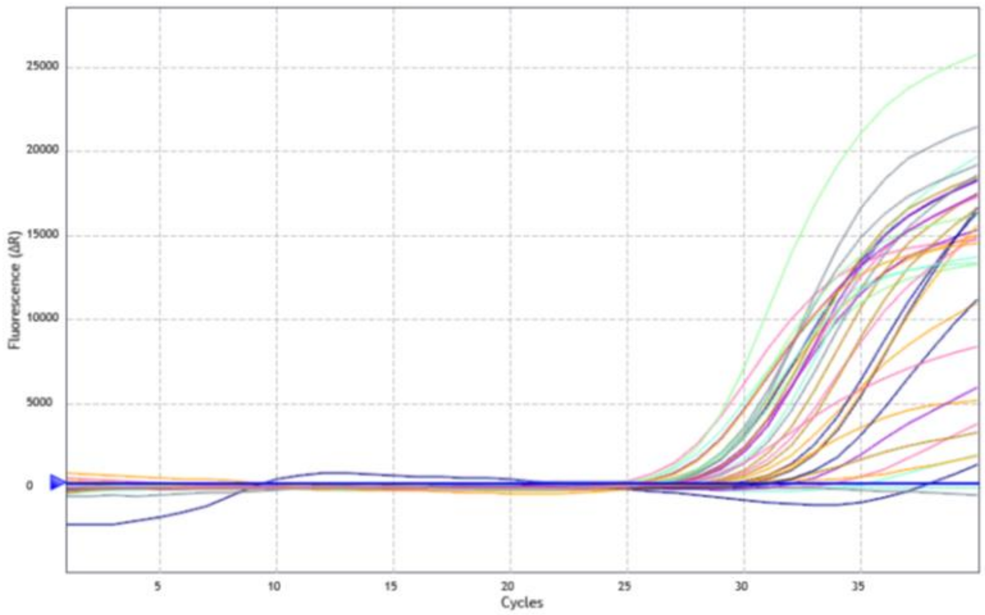

Fig. (S3a). Amplification curves of Methylated ABCG1 gene in patients of type 2 diabetes showing respective Ct values.

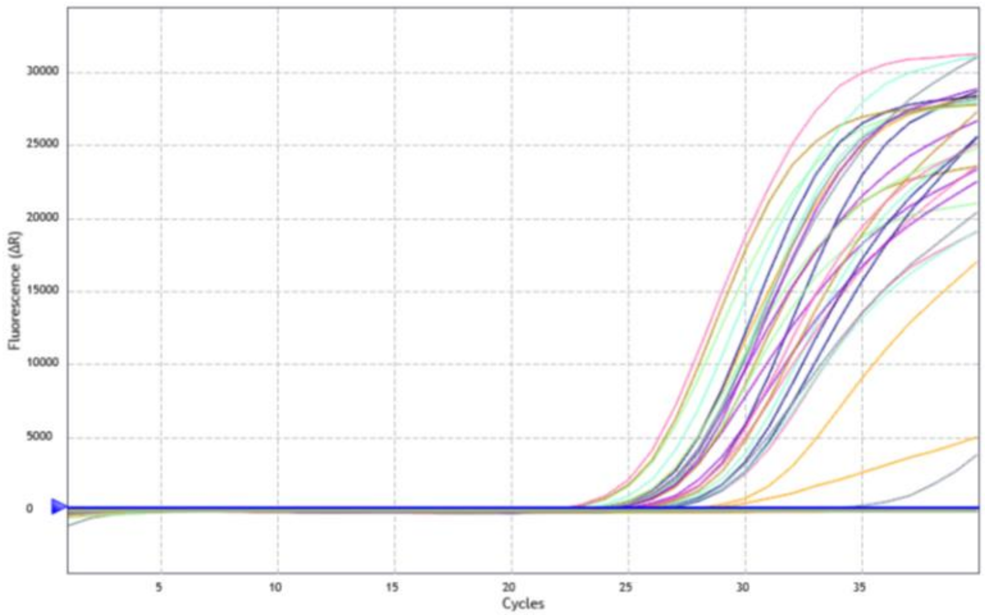

Fig. (S3b). Amplification curves of Unmethylated ABCG1 gene in patients of type 2 diabetes showing respective Ct values.

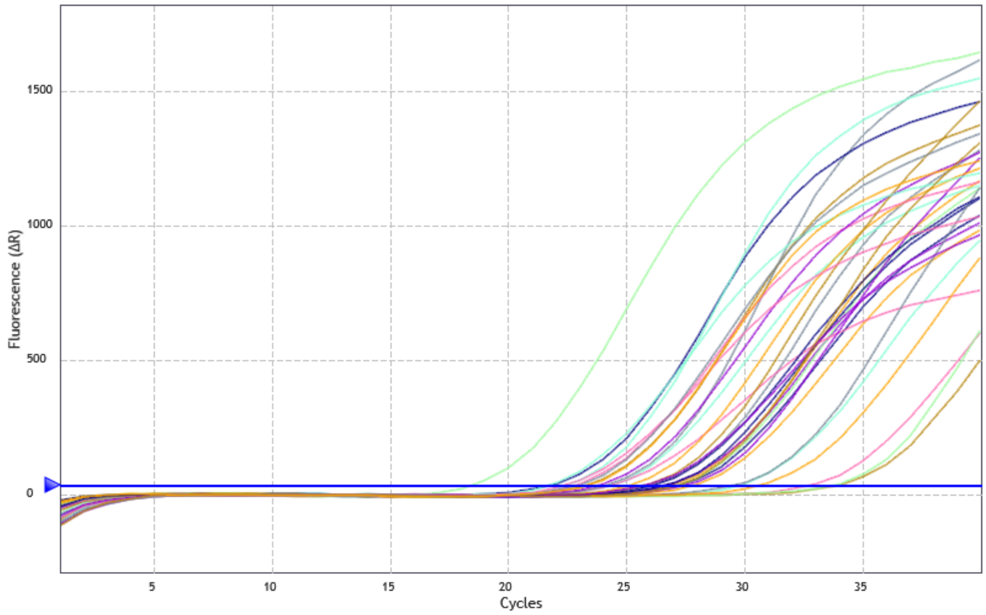

Fig. (S3c). Amplification curves of Methylated TXNIP gene in patients of type 2 diabetes showing respective Ct values.

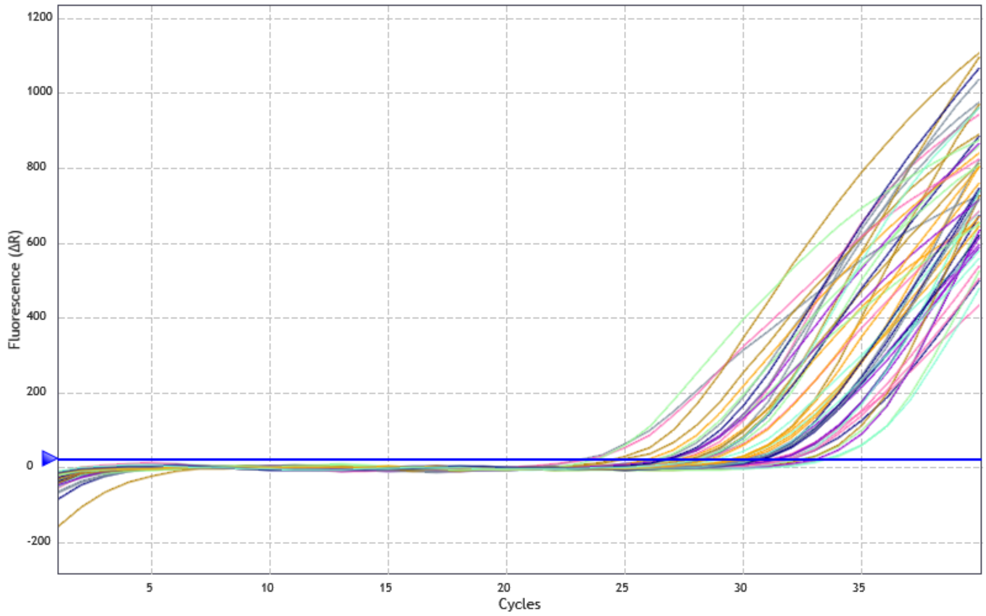

Fig. (S3d). Amplification curves of Unmethylated TXNIP gene in patients of type 2 diabetes showing respective Ct values.
